# A Prospective Study of Long-Term Regenerative Endodontics Outcomes of Necrotic Immature Permanent Teeth: An 8-Year Follow-Up

**DOI:** 10.3390/healthcare9121670

**Published:** 2021-12-02

**Authors:** Sawsan T. Abu Zeid, Ruaa A. Alamoudi, Osama S. Alothmani, Abeer A. Mokeem Saleh, Amna Y. Siddiqui

**Affiliations:** 1 Department of Endodontics, King Abdulaziz University, Jeddah 21589, Saudi Arabia; ralamoudi1@kau.edu.sa (R.A.A.); osalothmani@kau.edu.sa (O.S.A.); aasaleh@kau.edu.sa (A.A.M.S.); asiddiqui@kau.edu.sa (A.Y.S.); 2 Department of Endodontics, Cairo University, Cairo 12613, Egypt

**Keywords:** immature necrotic tooth, regenerative endodontics, treatment outcomes

## Abstract

For the management of necrotic immature teeth, regenerative endodontics offers the advantage of further root lengthening, thickening of dentin wall, and apical closure. This prospective study aimed to evaluate the long-term outcome of regenerative endodontics in immature necrotic permanent teeth. A total of 23 immature roots were medicated by triple antibiotic paste. After 21 days, bleeding was induced by over-instrumentation, and then mineral trioxide aggregate and coronal restoration were applied. Patients were scheduled for clinical and radiographic follow-up for 8 years. The radiographic changes of root dimensions were assessed using the ImageJ Plugin and statistically analyzed by Kruskal–Wallis test at a 95% confidence level. For qualitative evaluation, images were overlapped and analyzed using Photoshop software. All teeth were asymptomatic one month after the treatment. All teeth (*n* = 18) with preoperative periapical radiolucency showed complete resolution within 6–9 months. Recall rate at two, three, and eight years was 69.6%, 56.5%, and 34.8%, respectively. Continuous root development with a significant increase in root length and thickening of dentin wall accompanied by a significant decrease in apical canal diameter was seen at the end of the observation period (*p* < 0.001). In conclusion, the long-term outcome of regenerative endodontics revealed successful clinical and radiographic results with appropriate case selection.

## 1. Introduction

Dental trauma in young children can lead to pulp devitalization of immature permanent teeth and cessation of root development. Endodontic treatment of immature necrotic permanent teeth is challenging. The divergent radicular morphology, accompanied by lack of apical constriction and thin dentin, can lead to inadequate root canal disinfection and overfilling with a high risk of extruded gutta-percha [1]. The orthograde approach via apexification technique using either calcium hydroxide (Ca(OH)_2_) or mineral trioxide aggregates (MTAs) is the treatment of choice for such cases [2]. 

Apexification technique with long-term application of Ca(OH)_2_ has been used to induce a calcified apical barrier against which gutta-percha is condensed [2,3]. The main drawbacks of this technique are prolonged observation time and increased root fracture susceptibility [4]. Histologically, the dentin bridge formed after Ca(OH)_2_ apexification showed a porous “Swiss Cheese configuration” with a lack of a fluid-tight seal [5]. The introduction of MTA dramatically changed the management approach of immature necrotic permanent teeth [6]. The single appointment apexification technique using MTA apical plug demonstrated complete periapical healing with the formation of new apical hard tissue [3]. Yet, it did not provide root maturation [6]. Although both apexification techniques are successfully managing apical periodontitis associated with immature necrotic permanent teeth, they failed to improve the radicular length and canal width [4], leading to poor crown/root ratio and compromised tooth prognosis [7,8]. 

Regenerative endodontics has gained enormous attention over recent years [1]. Proposed by Nygaard-Ostby in 1961 [9], inducing bleeding through over-instrumentation introduces stem cells from the periapical tissue into the disinfected root canal and provides a scaffold that helps in the regeneration of dentin–pulp complex after interaction with growth factors released from radicular dentin [1,10]. Regeneration of the pulpal tissue offers an advantage of further root lengthening and thickening of the dentin wall with an apical constriction that increases fracture resistance [7,11]. It was suggested that the proper root canal disinfection can create a suitable environment for further tissue growth [7]. The literature is replete with studies reporting on the outcome of pulp regeneration. Most of the reported results are based on short-term observations [11,12,13,14,15,16]. A long-term outcome assessment is needed. This prospective study aimed to report the long-term clinical and radiographic outcomes of regenerative endodontic procedures of immature necrotic permanent teeth over a period of eight years.

## 2. Materials and Methods

### 2.1. Clinical Procedures 

Recruitment of 23 cases extended from September 2011 to February 2012 among patients seeking dental care at King Abdulaziz University, Faculty of Dentistry. Eligible participants were informed in detail about the procedures, postoperative care, follow-up, and alternative treatment options available to them. Inclusion criteria to be eligible for the study were as follows: the participant must have a carious or traumatized tooth with necrotic pulp (confirmed by cold and electric pulp tests (EPT); an immature root with open apex based on radiographic assessment; patients that were not allergic to medications and/or antibiotics (American Society of Anesthesiologists classification I or II). Exclusion criteria were as follows: necrotic teeth with closed apex; teeth with vital pulp confirmed by diagnostic tests (cold and/or EPT); teeth with suggestive signs of coronal fractures; teeth deemed non-functional or non-restorable; medically compromised patients.

A periapical digital radiograph was taken using a computerized digital image processing system (Macintosh Quadra 660 AV, Apple Computer, Ismaning, Germany) with a constant source-to-film distance of 5 cm and an object-to-film distance of 5 mm using Rinn’s film holder (Dentsply, New York, NY, USA), at 70 kV and 10 mA. Exposure time was automatically adjusted by the machine according to the examined area [17]. A digital radiograph using R4 software (Caresteam Dental LLC, Atlanta, GA, USA) was used to assess the preoperative root length up to cementoenamel junction (CEJ), the apical canal width, dentin wall thickness, and the presence or absence of periapical radiolucency. All included teeth were classified as Cvek stage III or IV [18]. A total of 20 patients fulfilled these criteria and were included in the study. 

Treatment was carried out in two visits [11,12,19] following the recommended protocol described by Banchs and Trope 2004, at the time of treatment [19]. In the first appointment, the tooth was anesthetized with 2% lidocaine with 1:100,000 epinephrine. After rubber dam application, access cavity and root canal irrigation with 10 mL of 2.5% sodium hypochlorite (NaOCl) were performed. Gentle irrigation using closed ends with a side-vent needle was penetrated and positioned 2 mm short from the root end. Then, the canal was flushed with 20 mL of saline and dried with paper points. Lateral dentin walls of coronal cavity were coated with a thin film of universal resin adhesive (Single bond universal adhesive, 3M^TM^ Dental, Neuss, Germany) using brush applicator then received 15 s of light cure, to avoid the dissemination of antibiotic paste to coronal dentin structure. Triple antibiotic paste (1:1:1 ratio of metronidazole, ciprofloxacin (Minapharm pharmaceuticals Co., Cairo, Egypt), and doxycycline (Pfizer Co., Cairo, Egypt)) was applied [11,14,20]. The tooth was then temporarily restored with IRM (Dentsply Sirona, Charlotte, NC, USA). 

The second appointment was scheduled after three weeks [11,14]. If the patients were asymptomatic, the tooth was anesthetized using 3% Scandonest without epinephrine (Scandonest 3% plain; Septodont, Saint-Maur-Des-Fosses, France), rubber dam was applied, and the antibiotic paste was gently removed using 10 mL 2.5% NaOCl irrigation, followed by saline irrigation. If the canal could not be dried due to exudation, the antibiotic paste was reapplied for another week. If the canal could be dried, over-instrumentation with a suitable K-file (Dentsply Maillefer, Ballaigues, Switzerland) was performed to induce intra-radicular bleeding. Bleeding was controlled to the level of cementoenamel junction (CEJ). After 15 min, as the blood clot has been formed, MTA (ProRoot MTA, Dentsply, Tulsa Dental, Johnson City, TN, USA) was carefully applied using double regular amalgam carrier (HuFriedy Co LLC, Chicago, IL, USA) over the blood clot at the level of CEJ, followed by a moistened cotton pellet. The tooth was temporarily sealed with IRM. An immediate postoperative radiograph was taken. On the following day, the coronal access was restored with light-cured composite resin (Filtek™ Supreme, St. Paul, MN, USA) after removal of the moist cotton pellet and MTA setting was confirmed.

Recall appointments were scheduled at 2 weeks, 1, 6, 12, 24, and 36 months or if the patient felt any symptoms. At each follow-up appointment, clinical examination was conducted including evaluation of the presence of pain/discomfort, swelling, sinus tract, response to cold tests and EPT, tenderness to percussion/palpation. Tooth mobility and probing depth were also recorded. The first radiographic assessment was carried out at 6 months of follow-up. Radiographic assessment was performed using a digital periapical radiograph as described earlier to assess any periapical changes, narrowing of the apical canal width, thickening of the root canal wall, and increasing in root length [11]. The success of regenerative endodontics was based on the following: a symptom-free functional tooth, radiographic evidence of resolution of periapical radiolucency (if present), and an increase in root development. Failure to achieve these goals was considered an unfavorable outcome. 

The inter- and intra-examiner reliability were performed. One author (S.T.A.Z.) was calibrated on selected radiographs and designated to evaluate all radiographs then rechecked by another author (R.A.A.). A repetition of radiographic assessment was performed after one month to determine the degree of agreement among and within the two independent examiners in regard to root length, apical canal width, and dentin wall thickness. Regarding intra-examiner reliability, the Kappa index was strong (κ = 0.91). The inter-examiner Kappa index of agreement between the examiners was calculated (κ = 0.86) which also indicated substantial to strong agreement.

Radiographs were analyzed quantitatively and qualitatively.

### 2.2. Quantitative Assessment

For quantitative assessment, the changes in root length, the diameter of apical canal width, and dentin wall thickness were assessed using ImageJ Plugin software (Java-based image processing program, version 1.44, 64-bit Java 1.8.0_112, National Institute of Mental Health, Bethesda, MD, USA). The plug-in tool (TurboReg, Biomedical Imaging Group, Swiss Federal Institute of Technology, Lausanne, Switzerland) was used to eliminate any dimensional error due to different X-ray angulations incorporated within the follow-up images [21]. All radiographic images were saved in JPEG format. A measuring scale was set on an immediate postoperative image based on a predetermined root length in a millimeter unit. Thereafter, root lengths, the thickness of the radicular dentin wall, and canal width were measured on each follow-up image at the same level as the preoperative image. Root length was determined as the average of 2 straight lines drawn from the CEJ to the radiographic root apex at the mesial and distal sides. The increase in root length (mm) was calculated. The thickness of the radicular dentin wall and the canal width were determined by using the straight-line tool, crossing the radiographic apical end.

### 2.3. Qualitative Assessment

**Figure 1:**
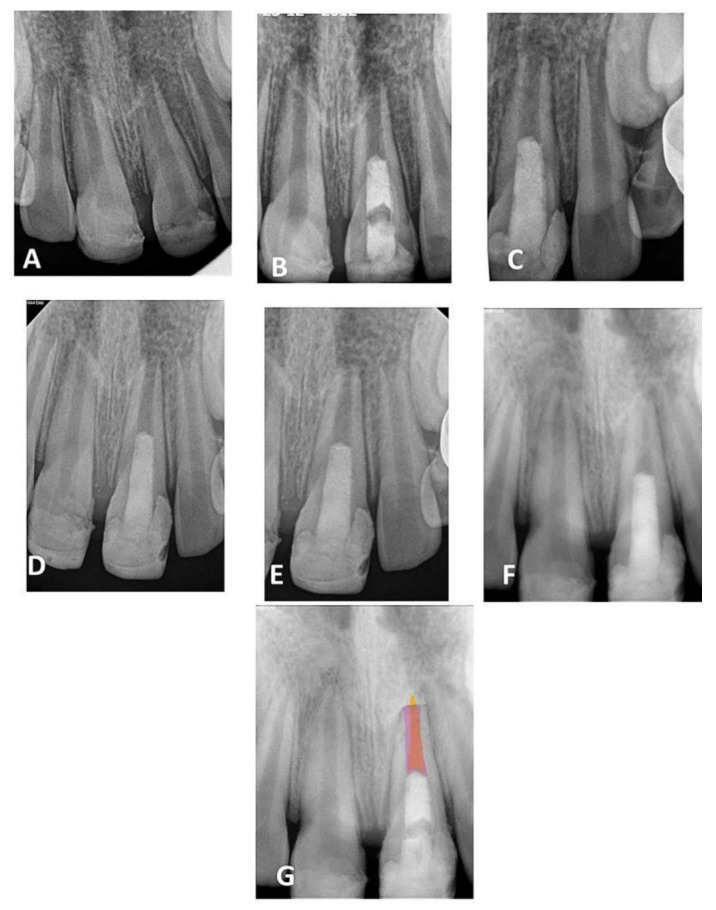
(**A**) Periapical radiograph of traumatized nonvital tooth #21 of a 9-year-old with wide-open apex; (**B**) immediate post-treatment radiograph showed MTA plug in the coronal third and composite restoration; (**C**–**F**) follow-up images after 6, 12, 24, and 36 months, respectively, showed consequence reduction in apical canal width, thickening of the radicular dentin wall, and increase root length; (**G**) overlapped periapical radiographs of immediate post-treatment and at 36 months of follow-up showed an increase in the root lengthen and apical closure after 36 months.

Qualitative evaluation was performed using Adobe Photoshop CS5 software (64 bit, Version 12, Sun Jose, CA, USA) to identify the dimensional changes of apical canal width and root length during follow-up periods. All images were collected and exported to Adobe Photoshop CS5 software (Figure 1). Using the lasso tool, the mode of images was changed to Red Green Blue (RGB) color and the root canal lumen of each follow-up period was traced and filled with a different color using the paint bucket tool [22,23]. The image of the immediate postoperative radiograph was traced, copied, pasted, and then moved to the image of each follow-up. At the opacity of 40%, the root canal lumen of the immediate postoperative image (pink color) was then overlapped with the lumen of each follow-up image to show the root canal dimensional changes (Figure 1G). The CEJ and the apical border of the MTA orifice barrier were used as fixed landmarks for overlapping the image. 

### 2.4. Statistical Analysis

Data were entered and analyzed using SPSS Statistical analyses (Version 20, IBM SPSS Statistics, Armonk, NY, USA). The normality of the data distribution was tested using Kolmogorov–Smirnov, three measurement data (root length, width of apical canal, and dentin wall thickness) were found not to satisfy the normal distribution. Hence, Kruskal–Wallis was used to evaluate the changes in root length, the width of the apical canal, and dentin wall thickness within the interval period. The level of significance was set at *p* < 0.05 and at the 95% level of confidence.

## 3. Results

**Figure 2:**
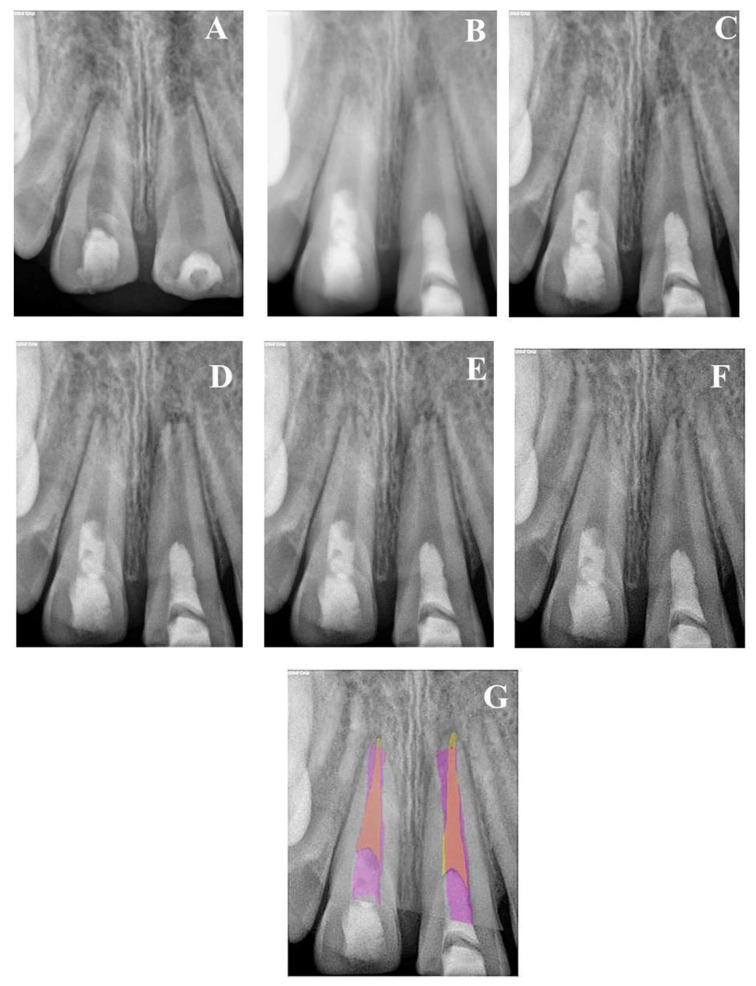
(**A**) Periapical radiograph of traumatized nonvital teeth #11 and 21 of a 9-year-old with wide open apices and periapical radiolucency; (**B**) immediate post-treatment radiograph showed MTA plugs in the coronal third of both teeth and composite restorations; (**C**) a follow-up photoradiograph after 6 months showed complete resolution of the periapical radiolucencies; (**D**–**F**) follow-up photoradiographs at 12 months, 24 months, and 8 years, respectively, showed narrowing of the apical foramen and further apical closure; (**G**) overlapped periapical radiographs of immediate post-treatment and at 8 years of follow-up showed significant changes in root length and width of apical canal.

**Figure 3:**
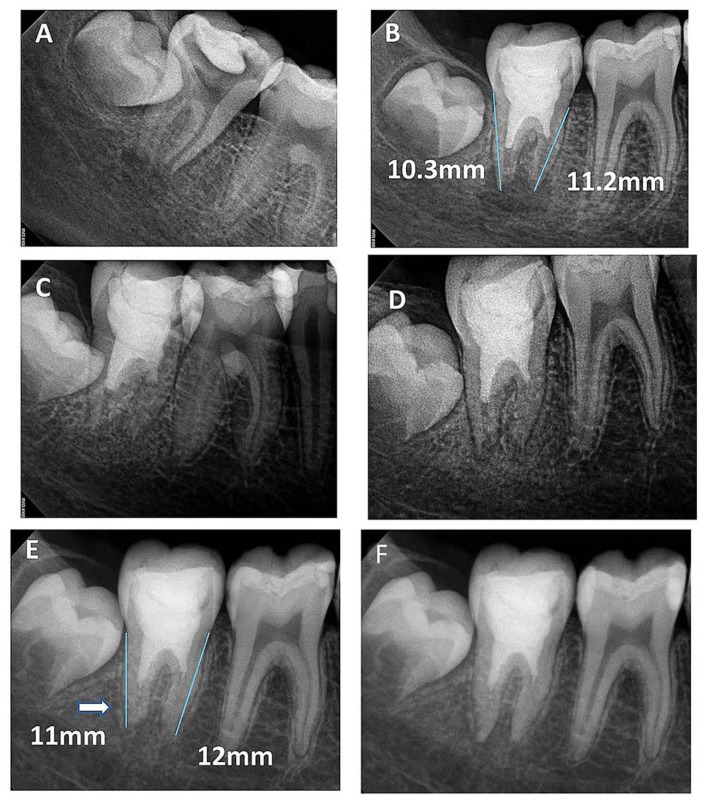
(**A**) Periapical radiograph of nonvital tooth #47 of a 9-year-old with wide open apex and periapical radiolucency; (**B**) immediate post-treatment radiograph showed MTA plug in the coronal third and composite restoration. The mesial root length was 11.2 mm and the distal was 10.3 mm; (**C**) a follow-up photoradiograph after 6 months showed a complete resolution of the periapical radiolucency; (**D**) follow-up photoradiograph at 12 months showed a decrease in apical canal width with evidence of mesial canal calcification; (**E**,**F**) Follow-up photoradiographs at 24 months and 8 years showed calcification of the apical distal canal and progressive mesial canal calcification.

**Figure 4:**
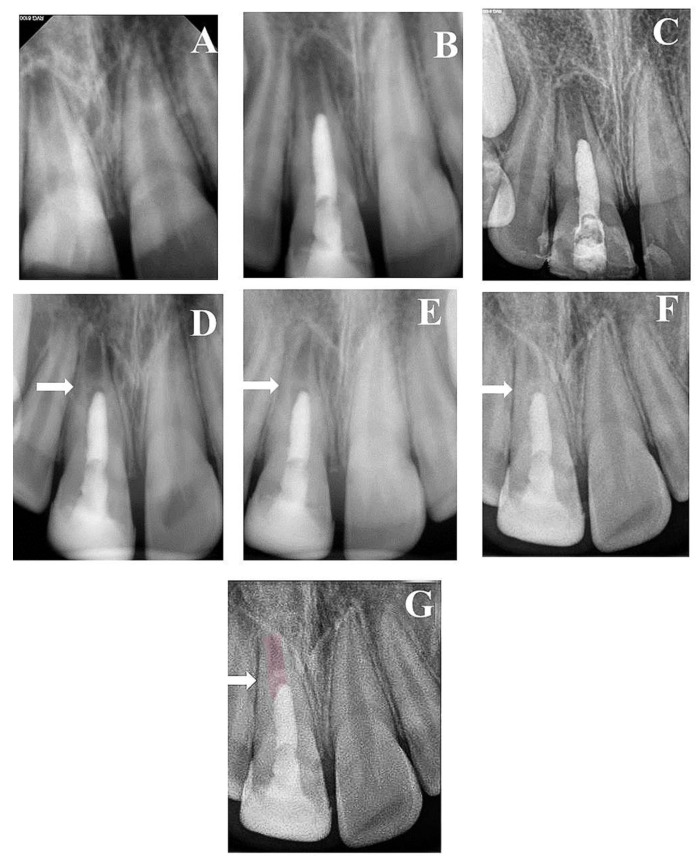
(**A**) Periapical radiograph of traumatized nonvital tooth #11 of an 11-year-old with wide open apex and periapical radiolucency; (**B**) immediate post-treatment radiograph showed MTA plug in the coronal third and composite restoration; (**C**) a follow-up photoradiograph after 6 months showed a complete resolution of the periapical radiolucency; (**D**) follow-up photoradiograph at 12 months showed evidence of radiopaque bridge (white arrow) under MTA orifice seal; (**E**) follow-up photoradiograph at 24 months showed narrowing in the apical canal area; (**F**) a follow-up photoradiograph at 8 years showed further reduction in apical canal width and thickening of canal dentin wall; (**G**) overlapped periapical radiographs of immediate post-treatment and at 8 years of follow-up showed significant changes in root length and width of apical canal.

A total of 23 teeth from 20 patients (17 (85%) males and 3 (15%) females) underwent regenerative endodontics procedures during the study period. All patients were healthy at an age of 9–13 years old. Patients’ demographic and preclinical characteristics are presented in Table 1. Most of the treated teeth were maxillary central incisors (*n* = 18), followed by two mandibular molars and one mandibular incisor, mandibular premolar, and maxillary molar of each. Preoperative periapical radiolucency was present in 18/23 teeth (78.3%). All included teeth were functional and asymptomatic one month after the treatment. They responded normally to percussion and palpation with normal mobility and probing. All treated teeth did not respond to cold tests or EPT through the 24 months. At three years of follow-up, 7/13 (53.8%) showed delayed response to EPT, while 4/8 (50%) responded at 8 years of follow-up. All 18 teeth with preoperative periapical radiolucency showed complete resolution within 6–9 months (Figure 2, Figure 3 and Figure 4). At 24 months of follow-up, 16/23 teeth were evaluated (recall rate = 69.6%). At 36 months of follow-up, 13/23 teeth were assessed (recall rate = 56.5%). At the eight-year follow-up, 8/23 teeth were examined (recall rate = 34.8%). At 12 months of follow-up, one mandibular molar (6.25%) showed evidence of root calcification (Figure 3D), and two maxillary central incisors (12.5%) showed the formation of the calcific barrier under MTA (Figure 4D). Two patients complained of tooth discoloration and were referred to the department of restorative dentistry for esthetic coronal restoration.

### 3.1. Quantitative Assessment

Median values of baseline and different follow-up periods regarding changes in the root length, the width of the apical canal, and dentin wall thickness are listed in Table 2. Root length and dentin thickness consistently increased over the observation periods, while apical canal width continued to decrease. At 24 months of follow-up, the increase in root length was insignificant (*p* = 0.164). However, the decrease in canal width and the increase in dentin thickness were both significant (*p* = 0.003 and 0.006, respectively). At the eight-year follow-up, there was a significant improvement in the three parameters, compared with baseline conditions (*p* < 0.001).

### 3.2. Qualitative Assessment

**Figure 5:**
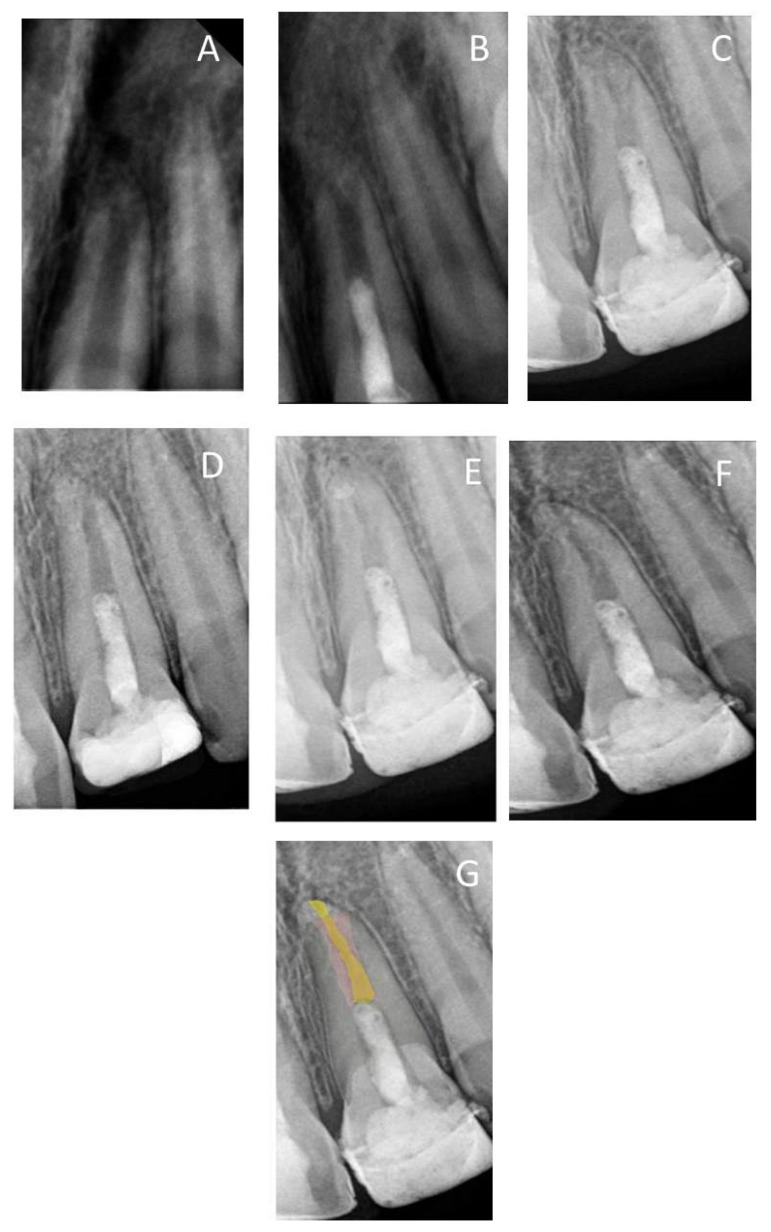
(**A**) Periapical radiograph of traumatized nonvital tooth #21 of a 10-year-old with wide open apex; (**B**) immediate post-treatment radiograph showed MTA plug in the coronal third and composite restoration; (**C**) follow-up photoradiograph after 6 months; (**D**–**F**) follow-up photoradiographs at 2, 3, and 8 years, respectively; (**G**) overlapped periapical radiographs of immediate post-treatment and at 8 years of follow-up showed a reduction in apical canal width, thickening of the radicular dentin wall, and increase in root length.

Radiographic examinations revealed progressive narrowing of the apical canal width, thickening of the radicular dentin wall, and an increase in the root length, as shown in the overlapped images of Figure 1, Figure 2, Figure 3, Figure 4 and Figure 5.

## 4. Discussion

Management of immature necrotic permanent teeth includes different treatment options aiming to induce apical closure [8]. Apexification technique, using either long-term Ca(OH)_2_ or MTA apical plug [1,3,6], proved successful outcomes. Yet, it does not allow complete root development, leading to thin brittle roots susceptible to fracture [24]. Pulp regeneration promotes root development and maturation of the apex by the ingrowth of de novo pulp-like tissue [11]. This study started with 23 cases of non-Saudi patients. During follow-up, the recall rate of the patients was progressively decreased to become eight cases (34.8%) at the 8-year follow-up. This was a result of patients’ address change and loss of contact numbers.

The regenerative endodontics approach is based on three major interactive components: (a) stem cells or undifferentiated mesenchyme cells capable to differentiate into fibroblasts, odontoblasts, and/or cementoblasts [11,13,25,26]; (b) a scaffold that allows cell growth and differentiation; (c) signaling molecules which are the growth factors that are capable of stimulating cellular proliferation and direct cellular differentiation. The induction of blood from the periapical area is a primary step in regeneration. This blood contains abundant undifferentiated stem cells and growth factors essential for root development [13] and acts as a scaffold that allows the attachment of stem cells [11,13].

The use of triple antibiotic paste in the current study was effective to eradicate polymicrobial flora present in the root canal system [27]. Various combinations of topical antibiotics may be used to disinfect necrotic infected root canals. It was suggested that the triple antibiotic paste composed of ciprofloxacin, metronidazole, and minocycline, as proposed by Hoshino et al. [28], is a favorable antimicrobial regimen for successful regenerative outcomes [12,13,29]. Moreover, other studies confirmed that a low concentration of triple antibiotic paste promoted the survival and proliferation of human stem cells of apical papilla [30,31]. Although many studies reported a high success rate with the use of antibiotic paste [19,32], the European Society of Endodontology recommended the use of Ca(OH)_2_ instead of antibiotic paste [32]. Doxycycline was used in our study instead of minocycline due to its unavailability [11,14,20]. Doxycycline was used in conjunction with metronidazole and ciprofloxacin because of its substantivity, as it can maintain antimicrobial action for a long time [33]. Several studies reported the application time range of triple antibiotic paste between 7 and 28 days [1,11,13,34,35]. Tooth discoloration occurred in two cases. The low incidence of discoloration could be attributed to the application of resin adhesive over coronal dentin, which prevented antibiotic paste seepage into the crown’s dentinal tubules. In the present study, the root canal was irrigated with a low concentration (2.5%) of NaOCl, and then the triple antibiotic paste was applied for 21 days, to ensure complete elimination of residual infection [11] and to provide a favorable environment for periapical healing and tissue regeneration [35]. This did not follow the recent American Association of Endodontists (AAE) recommendation which included the use of Ethylenediaminetetraacetic acid (EDTA) and saline in the irrigation protocol [36], as our study was carried out between 2011 and 2012, which is before the release of the AAE guide. Nevertheless, the lack of use of EDTA did not seem to hinder root development.

Previous case reports, case series, and clinical studies reported high survival and success rates with pulp regeneration [1,11,13,19]. According to the AAE 2016 [36], the success of regeneration is measured by three outcomes. The primary outcome is the elimination of the patient’s symptoms with complete periapical healing. The second outcome is the radiographic evidence of root development including an increase in root length, thickening of the dentin canal wall, and apical closure. The third outcome is regaining tooth vitality that is confirmed by sensitivity tests. A positive response to cold and/or EPT occurs in some cases [37] and could not be achieved in all cases, as it depends on the type of tissue filled the root canal and could indicate a more organized vital pulp tissue. In the present study, the positive response to EPT was established in seven (53.8%) and four (50%) cases at 3 and 8 years of follow-up, respectively. Previous studies reported that the positive response to cold test or EPT was documented in a few regeneration cases ranging between 13.3% [38] to 40% [39]. Due to this variability in response to sensitivity tests, it was decided that the main outcome of the present study will be based on a complete resolution of periapical lesion (if present) and progress of root development.

The results of this study revealed that the first outcome measured was fulfilled in all the cases. These results are consistent with other studies [1,19,40]. The second outcome was also promising. Changes in the radicular dentin length and width in the current study were emphasized by quantitative and qualitative evaluations of the radiographs. The quantitative measure was performed via ImageJ Plugin software, as described by various studies [1,11,41]. All root parameters were significantly improved with time. With regard to the root length, the present study showed an insignificant increase in the root length at two years (*p* = 0.164), whereas a significant increase was apparent at three and eight years (*p* < 0.001). A significant increase in the thickness of the dentin wall with a significant narrowing of the apical canal width was observed after regenerative endodontics procedures over all follow-up periods (*p* < 0.001). These findings were consistent with previous studies [1,6,11,13,19,25,26,29,35]. A qualitative evaluation was achieved in this study via overlapping the preoperative radiographic image with the follow-up images using Adobe Photoshop software. The overlapped images revealed a progressive increase in the root length and thickness of the dentin canal wall, whereas a decrease in the apical canal width was observed throughout all follow-up periods.

Several factors affect the outcome of regenerative endodontics. The patient’s age and preoperative apical canal diameter have a great impact on the outcomes. Estefan et al. [11] suggested that narrowing in the apical canal width is more predictable if the preoperative canal diameter is wider than 0.1 mm. In addition, significant increases in root length and thickness of the dentin wall have been associated with young patients between 9 and 13 years old. In the present study, the mean value of preoperative apical canal width was recorded at 1.25 ± 0.33 mm in 17 patients (74%) of 9–10 years old. The great success of this age may be attributed to the availability of abundant stem cells having regenerative potential [42].

The long-term follow0up we report in the current study was associated with sample size attrition—a well-recognized problem in such studies [43]. Variation in the sample size over different observation periods could explain why the increase in length was not significant at two years of follow-up, while it became significant later.

Chen et al. [35] described five radiographic responses to regenerative endodontics procedure. The present study showed only three. All treated cases showed apical closure with further root lengthening. One mandibular molar (6.25%) showed apical canal calcification of both mesial and distal root canals, which could be a result of a previous traumatic injury [44]. Two maxillary central incisors (12.5%) showed a radiopaque bridge under the MTA orifice seal, which may be attributed to the ingrowth of cementum-like tissue from the periapical area to pulp space [35].

As for future studies, long-term follow-up with a larger sample size is suggested for in-depth investigations. Moreover, studying the effect and outcomes of different bioceramic materials used in regenerative endodontics is essential.

## 5. Conclusions

In summary, regenerative endodontics procedure of immature necrotic teeth had a successful long-term outcome with clear evidence of maintaining apical health, continued root development, and complete apical closure. As shown in the present study, the evidence of resolution of a periapical lesion within the first year and the beginning of root development within the first two years can predict the progressive successful outcome of regenerative endodontic procedures.

## Figures and Tables

**Table 1:** Demographic data of the treated cases.

Case	Gender	Age in Years	Tooth	Etiology	Preoperative Root Length in mm	Preoperative Dentin Thickness in mm	Preoperative Apical Canal Width in mm	Length of the Follow Up	Resolution of Periapical Lesion in Month	Sensitivity Test at the 3rd Year Follow-Up
1	Male	9	11	Trauma	11.088	0.405	0.63	2 years	No lesion	NA
2	Male	9	21	Trauma	10.48	0.477	1.021	3 years	6	No respond
3	Male	9	11	Trauma	12.562	0.584	1.53	3 years	6	Respond
4	Male	9	21	Trauma	12.36	0.44	1.1	3 years	6	Respond
5	Male	10	11	Trauma	14.014	0.81	0.92	3 years	6	No respond
6	Male	11	21	Trauma	14.38	0.78	0.9	2 years	6	NA
7	Female	8	21	Trauma	10.01	0.36	1.02	3 years	6	No respond
8	Female	8	47	Caries	10.51	0.41	1.28	2 years	9	NA
9	Male	11	21	Trauma	10.34	0.79	2.32	8 years	6	Respond
10	Male	11	11	Trauma	10.02	0.51	1.85	8 years	6	Respond
11	Male	11	11	Trauma	10.375	0.56	1.68	8 years	6	No Respond
12	Male	11	11	Trauma	10.05	0.56	1.13	8 years	9	Respond
13	Male	11	11	Trauma	9.356	0.39	1.14	8 years	6	No respond
14	Male	13	31	Trauma	9.40	0.31	1.233	8 years	6	No respond
15	Male	11	21	Trauma	9.338	0.38	1.15	8 years	9	Respond
16	Male	9	35	Caries	9.40	0.31	1.16	8 years	6	Respond
17	Male	11	21	Trauma	9.401	0.36	1.23	12 months	6	NA
18	Male	10	21	Trauma	9.8	0.514	1.279	12 months	No lesion	NA
19	Male	10	11	Trauma	10.2	0.534	1.321	12 months	No lesion	NA
20	Female	8	37	Caries	10.477	0.78	1.214	12 months	No lesion	NA
21	Male	10	21	Trauma	9.56	0.419	0.923	12 months	No lesion	NA
22	Male	9	21	Trauma	10.023	0.465	1.13	12 months	6	NA
23	Male	8	16	Caries	9.401	0.402	1.24	12 months	6	NA

NA = Not applicable.

**Table 2:** Median of root canal dimensional changes (in mm) after regenerative endodontics procedure.

Follow-Up Time	Median at Each Follow-Up Time (mm)		
	Root Length	Apical Canal Width	Dentin Wall Thickness
Preoperative	10.36	1.14	0.48
6th Month	10.47	1.15	0.72
12th Month	10.64	1.28	0.74
2nd Year	10.79	1.29	0.76
3rd Year	10.92	0.65	0.86
8th Year	11.4	0.24	0.98

## Data Availability

The data presented in this study are available on request to corresponding author.
